# Addition of Rapamycin to Anti-CD3 Antibody Improves Long-Term Glycaemia Control in Diabetic NOD Mice

**DOI:** 10.1371/journal.pone.0067189

**Published:** 2013-06-24

**Authors:** Shira Perl, Jordan Perlman, R. P. Weitzel, Oswald Phang, Matthew M. Hsieh, John Tisdale

**Affiliations:** 1 Center for Human Immunology, NHLBI, NIH, Bethesda, Maryland, United States of America; 2 Molecular and Clinical Hematology Branch, NHLBI, NIH, Bethesda, Maryland, United States of America; La Jolla Institute for Allergy and Immunology, United States of America

## Abstract

**Aims/Hypothesis:**

Non-Fc-binding Anti CD3 antibody has proven successful in reverting diabetes in the non-obese diabetes mouse model of type 1 diabetes and limited efficacy has been observed in human clinical trials. We hypothesized that addition of rapamycin, an mTOR inhibitor capable of inducing operational tolerance in allogeneic bone marrow transplantation, would result in improved diabetes reversal rates and overall glycemia.

**Methods:**

Seventy hyperglycemic non-obese diabetic mice were randomized to either a single injection of anti CD3 alone or a single injection of anti CD3 followed by 14 days of intra-peritoneal rapamycin. Mice were monitored for hyperglycemia and metabolic control.

**Results:**

Mice treated with the combination of anti CD3 and rapamycin had similar rates of diabetes reversal compared to anti CD3 alone (25/35 vs. 22/35). Mice treated with anti CD3 plus rapamycin had a significant improvement in glycemia control as exhibited by lower blood glucose levels in response to an intra-peritoneal glucose challenge; average peak blood glucose levels 30 min post intra-peritoneal injection of 2 gr/kg glucose were 6.9 mmol/L in the anti CD3 plus rapamycin group vs. 10 mmo/L in the anti CD3 alone (P<0.05).

**Conclusions/Interpretation:**

The addition of rapamycin to anti CD3 results in significant improvement in glycaemia control in diabetic NOD mice.

## Introduction

Multiple medications have shown efficacy in preventing diabetes in the NOD mouse model of T1D, yet fewer have shown efficacy in reversing the disease after onset of overt hyperglycemia [1].

Among the immunomodulatory drugs that revert diabetes in the NOD mouse, anti CD3 has been studied extensively and has shown limited efficacy in clinical trials [2], [3], [4]. While NOD mice become insulin independent for long periods of time post treatment with anti CD3, humans have shown only temporary incomplete improvement in beta cell function. Possible explanations for the incomplete response observed in humans include a smaller residual beta cell mass, limited regenerative capacity of beta cells, or incomplete halt of the autoimmune attack. If the latter is the dominant cause of the incomplete responses observed to date, additional strategies aimed at tolerance inductionwarrant exploration. Indeed, the long-term efficacy of islet transplantation has also been limited by recurrent/persistent autoimmunity, and this barrier will also prove limiting with any new strategy involving the differentiation of pluripotent stem cells to a beta cell phonotype for transplantation.

We have previously demonstrated that rapamycin, an immunomodulatory agent, can induce operational tolerance in patients with sickle cell disease following non myloablative bone marrow transplant resulting in stable mixed chimerism, even in the absence of long-term immunosuppression [5] Rapamycin blocks the mTOR kinase which integrates multiple signals from the TCR (signal 1) as well as signals generated by costimulatory receptors (signal 2). Signal 1 activation of naïve CD4 cells in the presence of mTOR inhibition by rapamycin renders the cells regulatory T cells [6], [7]. While Valle et al have tested the combination of anti CD3 and Rapamycin in the hyperglycemic NOD mice and concluded that rapamycin breaks anti CD3 induced tolerance [8], their data is more consistent with temporary reversible beta cell toxicity from rapamycin administration.

We hypothesized that the addition of rapamycin to anti CD3 during the period of T cell recovery, when relative frequency of naïve CD4 T cells is increased, will improve glycaemia reversal rates and tested this approach in NOD mice with recent onset hyperglycemia.

## Materials and Methods

### Animals

Animal care and procedures were performed according to a protocol that was submitted and approved by the National Institutes of Health Animal Care and Use Committee (ACUC).

Six to eight week old NOD/Lt female mice were purchased from Jackson labs (Bar Harbor, ME, USA), and were maintained under specific pathogen-free conditions.

### Blood Glucose Monitoring

Beginning at 10 weeks of age, blood glucose was measured thrice weekly in the morning using aFreestyle Elite glucometer (Bayer, Germany).

A diagnosis of diabetes was made after two consecutive measurements of glucose >13.9 mmol/l. Once diabetes was confirmed the mice were assigned to one of two treatment groups, anti-CD3 alone or anti-CD3 with rapamycin (anti CD3+rapa).

### Treatment

All diabetic mice received a single injection of intraperitoneal (IP) non-Fc-binding anti CD3ε antibody (Fab’2 clone 145-2C11, Bio Express, West Lebanon, NH) at a fixed dose of 50 µg. Mice assigned to the combination treatment group received in addition a daily IP injection of rapamycin (Wyeth, DE) at 1 mg/kg for two weeks. Rapamycin was crashed and solubilized in carboxymethyl cellulose (CMC, Sigma) and a stock solution of 2.5 mg/ml. Rapamycin was further diluted in CMC immediately prior to I.P. administration at a dose of 1 mg/kg/day.

### Intraperitoneal Glucose Tolerance test (IPGTT)

Mice were fasted for 5 hr, with water ad lib, before receiving a single IP injection of 2 grams glucose per kilogram, 30% in 100 µl volume. Glucose tolerance was monitored via tail vein sampling at 0,15,30,60 and 120 minutes post glucose injection. IPGTT was performed between days 17–20 from the administration of the anti-CD3, at least 3 days from completion of rapamycin treatment.

A second IPGTT was performed after a rapamycin challenge to determine whether concurrent rapamycin administration affected glucose tolerance. The same mice (from both treatment groups) that had undergone the first IPGTT weretreated with 1 mg/kg of rapamycin for two consecutive days, and then the IPGTT was performed as described on the morning after the second dose of rapamycin.

### Statistical Analysis

To compare glucose values at each time point during the IPGTT unpaired *t* test was used.

The chi-square test was used to compare reversal rates between treatment groups.

Area Under the Curve (AUC) was calculated for glucose during glucose tolerance test using the Trapezoidal rule.
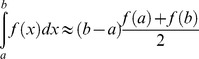



## Results

**Figure 1:**
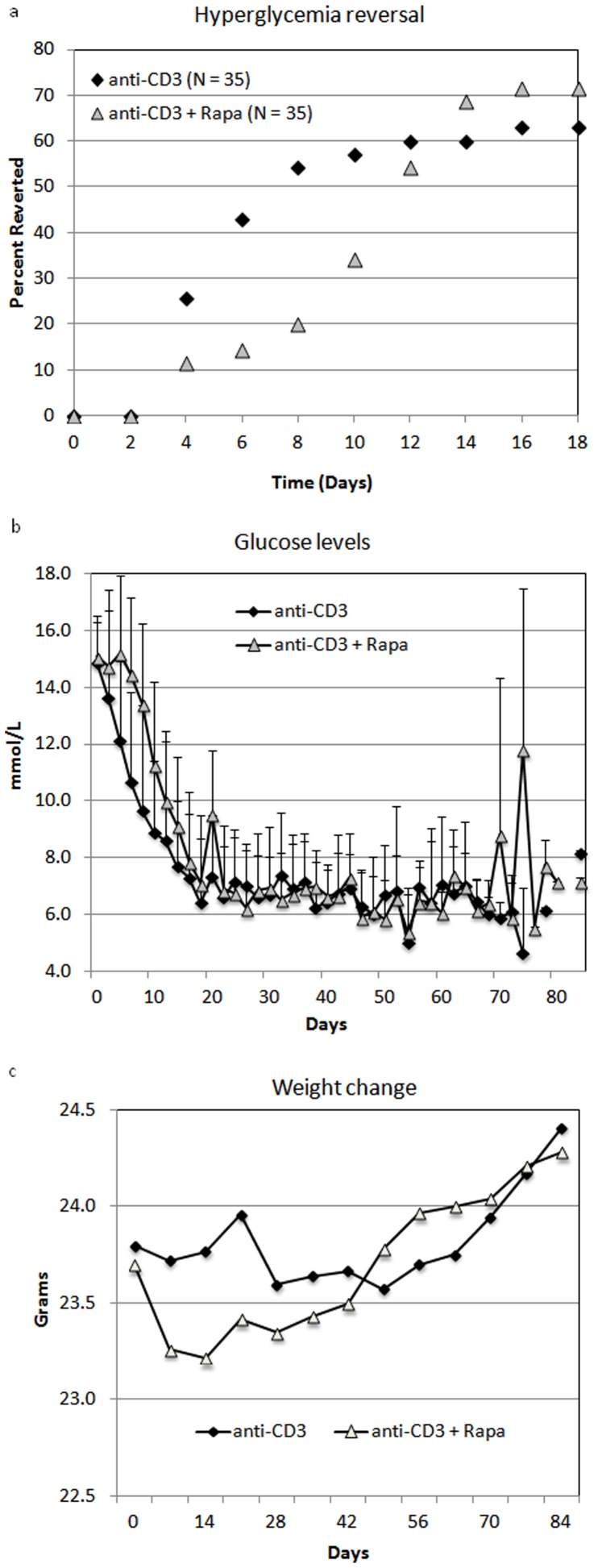
Anti-CD3+rapamycin vs. anti-CD3 monotherapy in diabetic NOD mice. Diabetic NOD mice were treated with either a single injection of 50 µg anti-CD3 alone (•) or a singleinjection of 50 µg anti-CD3 followed by 14 days of intraperitoneal rapamycin 1 mg/kg (gray triangle). (A) Percent reversal of diabetes over time (n = 35 for both groups, P = NS). (B) Changes in morning, non-fasting glucose levels over time. (C) Changes in weight over time.

To determine the optimal dose of anti-CD3 antibody by which greater than 50% of the mice revert to normoglcemia, we conducted a dose escalation study (doses of 18,25 and 50 mcg), which determined that a single IP injection of 50 mcg anti-CD3 antibody rendered approximately 60% of the mice euglycemic (data not shown). Based on these finding we randomized 70 hyperglycemic mice (glucose >13.9 mmol/L) to one of two treatment groups, single IP injection of 50 mcg anti-CD3 antibody followed by two weeks of daily IP injections of rapamycin 1 mg/kg for 14 days (anti-CD3+ rapamycin) or a single injection of IP 50 mcg injection only (anti-CD3 only). Percent reversal of hyperglycemia was higher in the anti CD3+rapamycin N = 25 out of 35 mice than the anti CD3 alone 22 out 35 mice reverted (P = NS, Fig. 1a). Non-fasting glucose levels did not differ between the treatment groups. Overall the time to reversal was shorter in the anti-CD3 only group compared to the anti CD3+ rapamycin.

Consistent with a pattern of slower reversal, glucose levels were higher in the anti-CD3+rapamycin group during the first two weeks while mice were actively receiving rapamycin, but normalized once the mice completed the treatment course (Fig. 1b). Weight gain was overall similar in both treatment groups with mice treated with rapamycin initially losing weight but later reaching equivalent weights (Fig. 1c).

To confirm the restoration of euglycemia, we performed an intraperitoneal glucose tolerance test on fasted mice from both study groups given the variability in the non-fasted state. Mice in the anti-CD3+ rapamycin group had superior glucose tolerance with lower fasting glucose levels and a smaller increase in blood glucose in response to the IP glucose injection (P≤0.05 at 0,30,60, and 120 minutes). Glucose area under the curve for the duration of the IPGTT were762 mmol/L/min and 1093 mmol/L/min for the combination and the anti CD3 alone respectively. These results of 30% better glucose control likely reflect higher insulin secretion and therefore higher residual beta cell mass.

To further test whether rapamycin is toxic to beta cells and directly cause higher blood glucose levels observed during the course of rapamycin and to explain the initial delay in diabetes reversal in the anti-CD3+ rapamycin group, we took mice from both treatment groups after completion of the treatment course, challenged the mice with two days of rapamycin, then performed IPGTT. Mice from both groups did worse while on rapamycin treatment compared to the first IPGTT. Again, mice that were initially treated with anti-CD3+rapamycin had a significantly better glucose tolerance than mice treated with anti-CD3 alone (P≤0.05 at 15,30,60, and 120 minutes).

## Discussion

**Figure 2:**
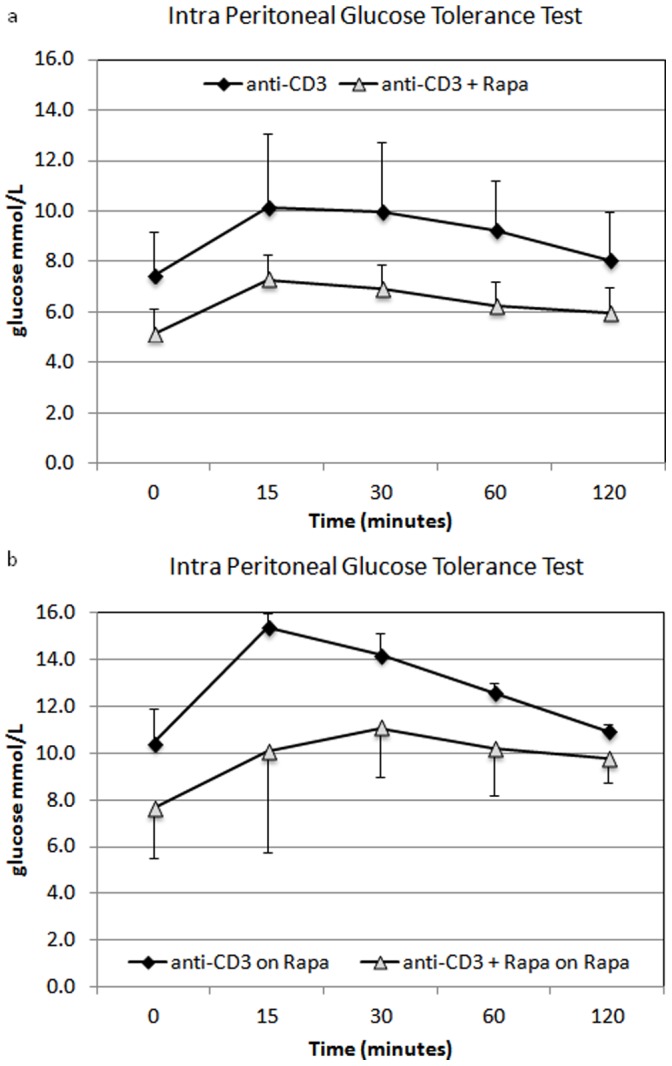
Intraperitoneal glucose tolerance test performed on fasted mice after completion of treatment course (day 17–20). (A) Mice who received anti-CD3 alone (N = 6) or followed by 14 days of rapamycin (N = 7) were injected IP with 2 grams/kg glucose solution and sampled for blood glucose levels. (B) Mice from both groups, anti-CD3 alone (N = 3) or in combination with rapamycin (N = 5) were challenged with rapamycin 1 mg/kg for two consecutive days. Glucose levels during an IPGTT done on day 3.

In this study we augmented the effects of anti-CD3 therapy on diabetes reversal rate and achieved better metabolic control by adding a short course of rapamycin. We tested our hypothesis in a sufficient number of overtly diabetic mice, consistent with recommendations for preclinical evaluation of therapies for T1D [1], using a reliable delivery method of rapamycin. Although rapamycin appeared initially to worsen glycemic control, our results support that rapamycin ultimately improves final beta cell function. The temporary increases in blood glucose (Fig. 1b) and weight loss (Fig. 1c) were fully reversed after discontinuation of rapamycin, the rapid reversal is not consistent with an immunologic effect. The mice that received rapamycin had significantly better fasting glucose levels and glycemic control in the IPGTT (Fig. 2a).

While hyperglycemia during rapamycin treatment in mice has been described previously [9], the rapid normalization of glucose levels and body weights after discontinuation of rapamycin has not yet been described. We observed a slower weight gain in the mice treated with rapamycin, possibly due to appetite suppression in the mice.

Valle et al [8] investigated a similar question but reached a different conclusion – rapamycin breaks anti-CD3 induced tolerance. We expanded their findings by showing that following a rapamycin challenge; glucose levels were higher in both groups of mice during the second IPGTT (Fig. 2b). These data, together with the reversible hyperglycemia and weight loss on rapamycin, suggest rapamycin exerts a direct toxic effect on beta cells. Although we did not investigate the direct immunologic effects of anti-CD3 and rapamycin, changes in immunologic tolerance should result in a much slower deterioration and much slower recovery of glucose control.

The main implication of our findings is that while rapamycin had an initial unfavorable effect on glycaemia during administration, long term it led to a significant improvement in overall glycaemia in an animal model of T1D. We believe that the overall improved glycaemia reflects higher residual beta cell mass and if achieved in a human clinical trial would be reflected by the gold standard, an improved c-peptide response to a mixed meal tolerance test. [10] Therefore a brief course of rapamycin in conjunction with other immunomodulatory regimens, including but not limited to anti-CD3, is a rational strategy that deserves testing, especially since evidence from other clinical settings support its immunomodulatory effects. The worsening of blood glucose levels with rapid reversal implicates rapamycin to have a direct causal relationship to beta cells. Nonetheless, rapamycin monotherapy administered to six patients with T1D awaiting islets transplantation did not affect glycemia control as measured by HbA1c and C-peptide levels. [11] Although rapamycin has benefited patients with sickle cell disease receiving allogeneic hematopoietic stem cell transplantation, the ability of rapamycin to induce tolerance to beta cell antigens in the setting of autoimmunity in a similar manner remains to be elucidated.
